# The Effect of Heart Rate on Jump-Shot Accuracy of Adolescent Basketball Players

**DOI:** 10.3389/fphys.2018.01065

**Published:** 2018-08-03

**Authors:** Johnny Padulo, Pantelis T. Nikolaidis, Drazen Cular, Antonio Dello Iacono, Stefano Vando, Maria Galasso, Dario Lo Storto, Luca P. Ardigò

**Affiliations:** ^1^Faculty of Psychology, eCampus University, Novedrate, Italy; ^2^Faculty of Kinesiology, University of Split, Split, Croatia; ^3^Department of Physical and Cultural Education, Hellenic Army Academy, Athens, Greece; ^4^Zinman College of Physical Education and Sport Sciences, Wingate Institute, Netanya, Israel; ^5^Department of Neurosciences, Biomedicine and Movement Sciences, School of Exercise and Sport Science, University of Verona, Verona, Italy

**Keywords:** basketball, bpm, fatigue, motor control, task performance and analysis, team sports

## Abstract

Basketball is a team sport, where fundamental skills – fundamentals – are key determinants for success. Jump-shot (JS) is a basketball fundamental used frequently during game. It is interesting to spread light on the relationship between effort intensity and JS ability. Study aim was to investigate different heart rates (HRs) effect on JS accuracy (JS%) in 22 male youth (15.7 ± 0.9 years) players. Experimental sessions consisted of 10 JSs from five spots 5 m from basket at three different HRs: rest (0HR) and after warm-up (50% [50HR] and 80% maximal HR [80HR]). Analysis of variance showed differences in JS% over sessions (42.27 ± 14.78% at 0HR, 38.18 ± 10.53% at 50HR, and 30.00 ± 16.62% at 80HR; *P* = 0.018). Least significant difference test did not show any significant difference between 50HR and 0HR JS% (*P* = 0.343), while 80HR elicited significantly lower values with respect to both 0HR (*P* = 0.006) and 50HR (*P* = 0.049). Study provided practical indications on maintaining high JS%: preliminary warm-up (even if injury-protecting) does not improve JS%, because between 50HR and 0HR difference was not significant; and 80HR significantly decreases JS%. Therefore, to maximize JS scoring players have to rest as much as possible during game-play pauses, and coaches should manage timeouts and substitutions accordingly, especially during final minutes of close games.

## Introduction

Basketball is a team sport, where performance depends on physical, physiological, psychological, technical and tactical characteristics. Among these characteristics, an increased scientific interest has been observed in the profiling of physical and physiological characteristics with regards to age and playing position (Nikolaidis et al., 2014, 2015). Accordingly, several studies have been conducted recently to develop sport-specific exercise tests (e.g., including uni- or multi-directional repeated sprints) in order to match the metabolic demands and activity patterns of basketball (Attene et al., 2015b, 2016; Padulo et al., 2015b, 2016; Zagatto et al., 2017), whereas other studies have examined the effectiveness of sport-specific training interventions (Attene et al., 2014, 2015a). Although the physical, physiological and psychological characteristics, tactical and nutritional aspectis, commonly considered as key components for successful basketball performance, have already been investigated, less information is available on technical skills, such as the shooting task (Padulo et al., 2015a; Ardigò et al., 2018). It has been supported (Okazaki et al., 2015) that the ability to shoot a jump-shot (JS) is a major component of sport performance. Moreover, it has been shown that players perform shooting more often under conditions of low-pressure and fast movements (Csapo et al., 2015). Professionals can shoot from longer distances than their amateur counterparts, and perform more team tactics in order to be in position where, likely, a relatively lower defensive pressure exists (Ibáñez et al., 2009). With regards to playing position, point guards and power forward perform shooting more frequently and successfully in free-throw (FT) and two-point shots, and point guards shoot more frequently successfully in three-point shots (Ortega et al., 2006).

The variables that affect ball trajectory have been well studied (Okazaki et al., 2015). When deciding to shoot, players consider four factors: physical defensive pressure, rebounding issues, defensive balance, and shooting distance (Llorca-Miralles et al., 2013). Time remaining before the shot clock expires might be another factor (Skinner, 2012). Shooting accuracy can be influenced by defensive intensity, and to a lesser degree by how many defenders are guarding the shooter (Csapo and Raab, 2014).

The abovementioned studies on factors influencing shooting performance have improved the scientific understanding about the key requisites targeted as fundamental for shooting performance accuracy. However, less data exist about the role of fatigue, induced by different exercise intensities, on JS%. Just as a reference, in the 2015–16 season (regular season and playoffs) the NBA champions Cleveland Cavaliers shot – contested by players from the opposing team – JSs from a distance of between 16 and 17 feet (between 4.88 and 5.18 m) in the first and last 2 min of the regular quarters with JS percentages (JS%) of 44.3 and 28.3%, respectively^1^. Clearly, directly from the above reference, it is not possible to clear which is the cause of the JS% decrease, be it fatigue, game intensity, time pressure… However, the JS is surely a popular basic shot that might be used in various conditions during a match (e.g., after continuous match-play or after a time-out). Information concerning the role of fatigue on JS% might be of practical interest for a large number of strength and conditioning coaches. As there have been no previous studies on the effects of exercise intensity on JS%, researchers might employ such data as reference for future research on this team sport. Moreover, strength and conditioning coaches could apply this information to prescribe proper training regimes to optimize shooting performance.

Basketball became one of the most popular team sports worldwide, but relatively few papers have been published so far about the determinants of shooting accuracy despite its great relevance for successful performance. Present study wished to provide field information on the relationship between fatigue and shooting ability and practical knowledge for players’ skills development trainers. Consequently, the objective of this study was to investigate the role of different heart rate (HR) conditions on JS%. We adopted the research hypothesis that an increased HR would result in decreased JS%.

## Materials and Methods

### Participants

Twenty-two adolescent male basketball players (age 15.7 ± 0.9 years; height 179.0 ± 6.9 cm; mass: 66.0 ± 9.6 kg; BMI 20.6 ± 2.0 kg/m^-2^; training experience 8.4 ± 3.0 years), players of 7 Laghi Gazzada team, volunteered for the present study. All participants were training regularly during weekdays and were playing official match every weekend. To be eligible for this study, a participant should have attended a minimum of 85% of all team’s exercise programs, had a valid sport medical certification and did not report any illness or injury. This study was performed considering the recommendations of the Code of Ethics of the World Medical Association. The protocol was approved by the University of Split Ethics Committee. All participants’ parents/guardians provided written informed consent in accordance with the Declaration of Helsinki.

### Protocol

Participants were instructed to abstain from drinking alcohol or beverages containing caffeine for 24 h, and did not eat for 3 h, prior to testing, in order to decrease their possible effect on the testing outcome. Each participant performed all trials in the same time period and under the same climatic conditions of the testing days (15:00–17:00, 22.5 ± 0.6°C temperature, and 59.2 ± 2.4% relative humidity), to minimize any effect of circadian variation. All tests were carried out on an indoor basketball court.

**FIGURE 1:**
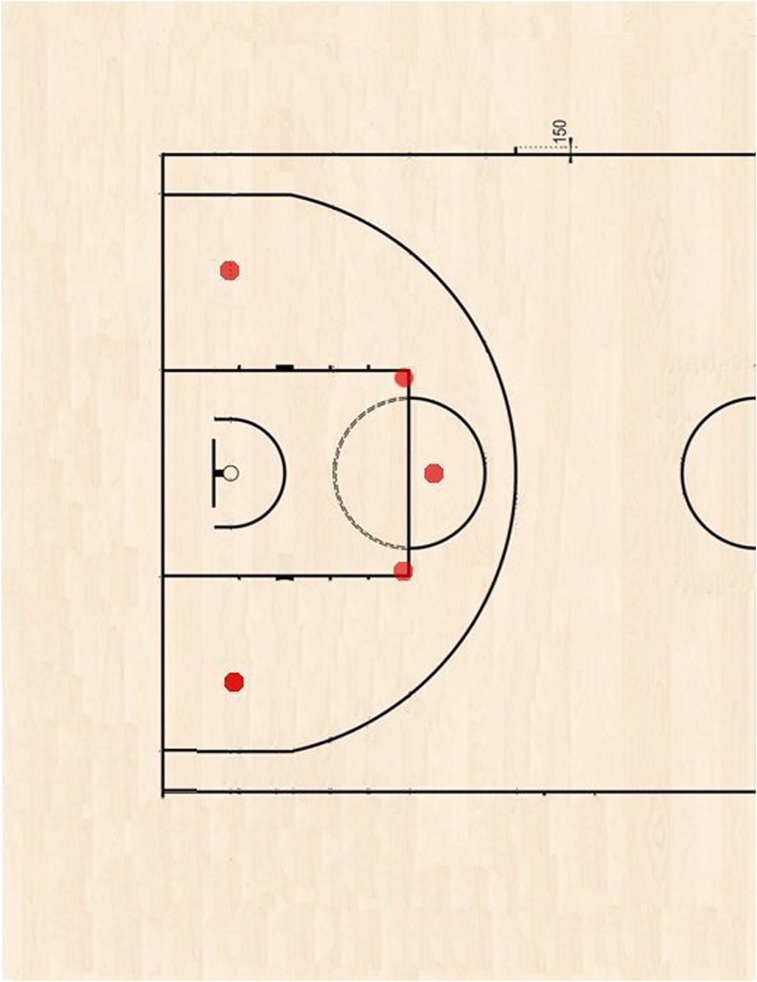
Jump-shot spots placement on half-court.

This experimental study was approached through a crossover observational design (i.e., all players shot their JSs over different sessions in different days). We used HR as the independent variable and JS% as the dependent variable. In the first session, the participants carried out a Yo-Yo Intermittent Recovery test level 1 (Yo-Yo IR1; Castagna et al., 2008) to measure maximal heart rate (HR_MAX_). One week later, the participants executed three randomized shooting testing sessions with a 1-h rest between two consecutive sessions. All sessions included 10 consecutive JSs (regular ball – Molten gf7, 600 gr.) from five different commonly chosen field spots, at a distance of 5 m from the (regular) basket (**Figure 1**, two counter-clockwise laps), at self-selected game-play pace, at different HRs: at rest without warm-up (0HR), immediately after warm-up with HR at 50%HR_MAX_ (50HR; i.e., a reasonable post-warm-up HR value; Garrett and Kirkendall, 2000), and 80%HR_MAX_ (80HR; i.e., a reasonable actual play HR value; McInnes et al., 1995). Two balls were used, and two rebounders ensured an accurate ball pass to the shooter at each different spot. The shooting distance was kept constant in order to eliminate its specific effect on shooting kinematics (Miller and Bartlett, 1996). The same procedure was repeated 1 week later to evaluate the measures’ reliability. Each HR (continuously monitored with Cardio-SuuntoTM) was achieved by increasing the intensity of the warm-up shuttle running (15 + 15 m with 180° changes of direction) chosen as an ecological way to simulate fatigue provided by game-play. Briefly, the participants needed to run on average 540 and 1620 m to achieve 50 and 80%HR_MAX_, respectively.

### Statistical Analysis

Descriptive statistics (mean ± SD and SE) were calculated for the Yo-Yo IR1, and the 0HR, 50HR, and 80HR (effective %HR_MAX_ and measured JS%, i.e., successful JSs/total JSs%) test results, to present the data. Statistical analyses were carried out using SPSS 17.0 (SPSS Inc., Chicago, IL, United States). Normality of the data was tested with the Shapiro-Wilk test and homogeneity of variances was examined using the Bartlett’s test. Intra-class Correlations Coefficient (ICC) estimated the reliability of the 0HR, 50HR, and 80HR tests. ICC evaluation *criterium* is: <0.4 poor, 0.4–0.75 fair to good, and >0.75 excellent (Fleiss, 1991). Furthermore, a one-way within-participants analysis of variance (ANOVA) examined differences among the three test levels (0HR, 50HR, and 80HR) with the *post hoc* Least Significant Difference test (LSD). Cohen’s *d* effect sizes (ESs) obtained in each statistical analysis were shown and interpreted as proposed by Hopkins^2^, with ES < 0.2 considered as trivial, 0.2–0.5 small, 0.6–1.1 moderate, 1.2–1.9 large, and >2 very large. Statistical significance was set at a *P* ≤ 0.05.

## Results

**FIGURE 2:**
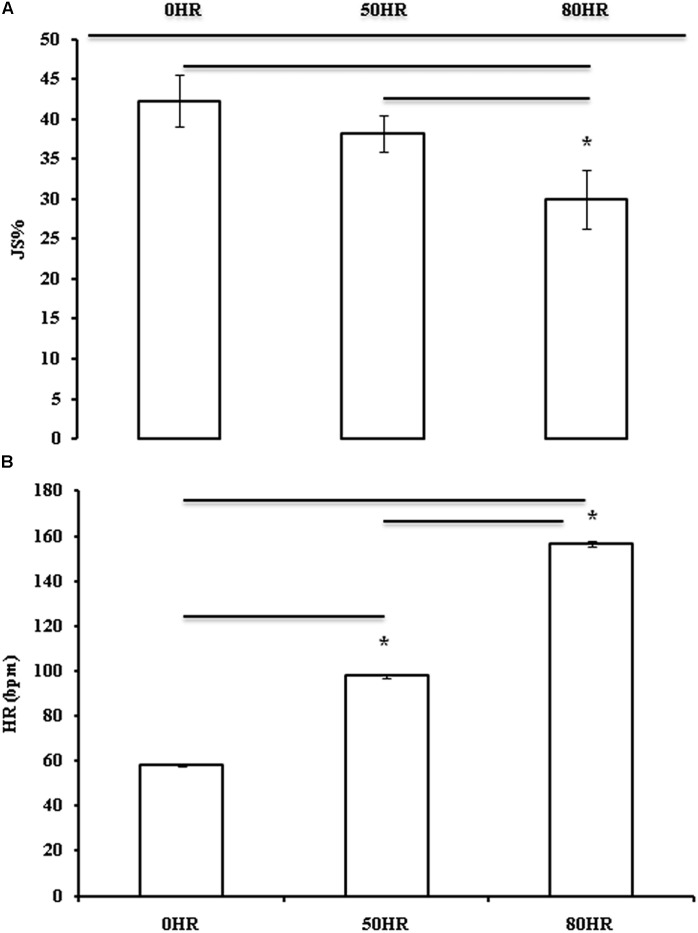
Percentage of jump-shot success (JS%), top **(A)** and heart rate [HR (bpm)], bottom **(B)**, as Mean ± SD, over the three different testing conditions (at rest, 50% maximal heart rate, and 80% maximal heart rate as 0HR, 50HR, and 80HR; *n* = 22). ^∗^*P* < 0.05.

The ICC demonstrated a good reliability at 0HR (0.89), 50HR (0.92), and 80HR (0.95). Yo-Yo IR1-induced HR_MAX_ was 196.8 ± 6.6 bpm, corresponded to a total distance of 1724 ± 671 m, and an end running speed of 18.3 ± 1.9 km/h. ANOVA showed differences of HR among the three applied exercise conditions (**Figure 2**; *F*_(1,20)_ = 4775.001 with *P* = 0.000): 0HR (58.3 ± 1.9 bpm), 50HR (97.8 ± 3.1 bpm), and 80HR (156.0 ± 4.9 bpm). Each shooting session lasted less than 1 min and did not contribute to increase starting HR more than 5%. Similarly, ANOVA showed differences of JS% over the three exercise conditions (**Figure 2**; *F*_(1,20)_ = 4.257 and *P* = 0.018), with a total of 660 JSs shot. JS% in the three exercise conditions was 42.27 ± 14.78% at 0HR, 38.18 ± 10.53% at 50HR, and 30.00 ± 16.62% at 80HR. The LSD test did not show any significant difference between 50HR and 0HR JS% (-10%, ES = -0.28, small, and *P* = 0.343), whereas 80HR elicited significantly lower values of JS% compared to 0HR and 50HR (-29 and -21% with ES = -0.83 and -0.78 (moderate), with *P* = 0.006 and *P* = 0.049, respectively).

## Discussion

In the present study the effect of exercise intensity on JS% was examined. Three conditions of exercise intensity were considered: rest (0HR), warm-up (50HR), and exercise simulating the average intensity of a basketball game (80HR). The main findings were that (a) JS% was lower in the 80HR than in the 0HR (-29%) and 50HR (-21%) conditions, and (b) no difference between 50HR and 0HR was observed.

To the best of our knowledge, the present study is the first to specifically examine the variation in JS% induced by exercise intensity. These results are in line with those of previous research on the effect of exercise intensity on FT (Padulo et al., 2015a; Mokou et al., 2016) and three-point shot (3S) accuracy (Ardigò et al., 2018), which showed a significant reduction in shot accuracy at 80HR compared to 0HR and 50HR. Thus, exercise intensity exerted a similar effect on the 3S, JS, and FT, yet, a difference can be identified between the 3S and JS compared with the FT. Namely, FT is not a game-play flow-driven basketball fundamental, given that it is basically shot without any defence pressure and during a game-play pause. In contrast, the 3S and JS are fundamentals performed throughout a much longer and complex game-play action, potentially involving the contribution of the entire playing team (e.g., by means of a plan), and with the opposition of one or more defenders. Therefore, game-play pace surely has some effect on 3S and JS performance, and real game-play study simulation may not be as effective here as in the FT simulation.

However, our outcomes – simply in terms of exercise intensity effect on JS% – are similar to those shown by previous studies on the effect of fatigue on passing accuracy (Lyons et al., 2006; Ahmed, 2013). Although the designed fatiguing protocols in these studies were different from the current study’s protocol (i.e., gym machines-based vs. intermittent shuffle running), the decrease in technical skill accuracy ranged from 15 to 30%, thus similar to that observed about JS%. To date, the effect that fatigue has on the performance of the basketball JS is not clear. From a biomechanical viewpoint, an appropriate whole body movement organization – from the lower body segments, through the trunk, and up to the ball-releasing segment of the hand-fingers complex, is required for the development of a shooting movement pattern that results in a successful shot. The JS represents a complex sequence of proximal to distal force and moment interrelations, with the goal of transferring energy from the lower extremity and trunk up and through the upper extremity kinetic chains. Finally, the upper extremity must effectively control and transfer these forces in order to sustain an optimal and accurate action. Therefore, a possible explanation of the deteriorating effect of fatigue on shooting accuracy might be the alterations of some kinematic characteristics occurring in the involved movements (whose specific description goes beyond the aim of this study). The sensorimotor system is responsible for providing the awareness, coordination, and feedback to maintain intra-joint stability and inter-joint coordination during complex movements (Lephart et al., 2000; Gheller et al., 2015). Evidence indicates that the sensorimotor system may be compromised by fatigue. Fatigue seems to hamper the sensorimotor system function in a way similar to joint injury. As a consequence, sensory motor deficits may result in the inability to appreciate and maintain the ideal mechanics that should accompany a prolonged shooting task (Forestier and Nougier, 1998). It has been shown that optimal timing, sequence, and amplitude of the muscle activation and joint movements are required for JS-like shot performance, even when the task is performed by different subjects (Forestier and Nougier, 1998). Moreover, some movement constraints (e.g., constraining the trunk segment) only slightly disrupt the pattern of variables used to describe coordination (e.g., muscle activation; Eloranta, 1996). Indeed, breakdowns in shooting mechanics may represent reasonable limiting factors for successful performance (Okazaki et al., 2015). Accordingly, studies that analysed the effect of fatigue on motor skills such as the vertical jump (Rodacki et al., 2002) revealed that when the primary muscles responsible for the jumping movement were fatigued, the movement resulted in greater involvement of the synergistic muscles. Thus, these results suggest that fatiguing the muscles involved in basketball shooting (e.g., fatiguing the lower limbs that perform the jump or the upper limbs that perform the ball release) may result in a decline in the player’s level of performance. For instance, it has been shown that fatigue induces a decrease in the height of the shoulder axis and of the wrist (Erculj and Supej, 2009). The underlying physiological mechanisms leading to the decrease in JS% at 80HR due to the fatiguing protocol may be attributed to various factors related to the muscle and blood metabolites. From a methodological perspective, the fatiguing protocol designed in our study to induce the targeted physiological responses (e.g., 80HR) implied a setup including intermittent low/high-intensity shuffle running bouts similar to those included in the Yo-Yo test (Castagna et al., 2008). Therefore, performing shuffle running repeatedly, with the inclusion of acceleration, deceleration, and change of direction actions, may have increased the chances for the players to incur a short-term fatigue-related adaptation that, in turn, may have altered the mechanics of the JS performance and hence the scoring accuracy.

A determining factor worth considering for the interpretation of the results of our study is the experience level of the involved population. Previous studies comparing the movement patterns of the FT and JS across players of different levels have identified an inter-individual variability, with technical performances being more variable in beginner players and less evident in experienced ones (Button et al., 2003; Okazaki et al., 2007). Specifically, novice players seem to constrain the degrees of freedom of their joint movements during the release phase of JS (Temprado et al., 1997), in order to make straightforward the controlling tasks imposed on the central nervous system during this phase (Newell and Vaillancourt, 2001; Okazaki et al., 2007). In turn, this constraining organizational pattern of motion prevents the beginner player from using a wider range of choices that have been shown in the game-play of experienced players. Consequently, sport experience is an aspect that might influence inter-individual differences when performing a JS under fatigued conditions. Hence, the evident greater variability observed among novice athletes in performing technical skills associated with fatigue-induced impairments on shooting accuracy may have produced a cumulative negative effect on the JS performance, as reported in our data in the 80HR condition. On the other hand, the age and sport experience (∼16 and ∼8 years, respectively) of the basketball players who represent the sample of this study may also constitute a specific limitation of the findings, given that the conclusions should be translated with caution to other age and performance groups. A previous investigation on the effect of exercise intensity on passing accuracy in basketball reported that passing performance decreased with increasing fatigue, in both novice and expert basketball players. However, the expert players showed less deterioration of performance (Lyons et al., 2006). Players’ young age and sport experience might have impacted study results in terms of specific age and training dependent heart responses (Francavilla et al., 2018). Another potentially confounding factor we neglected was players’ specific psychological genes-driven answer to study protocol (Sessa et al., 2011; Petito et al., 2016).

Further study limitations are the following ones. We focused as metabolic power proxy only on pre-set HR values and not on Karvonen’s HR reserve ones (Karvonen et al., 1957) and/or rates of perceived exertion (Borg, 1982). Our testing conditions were very controlled, whereas real game-play might be by its nature chaotic with different level opponents, many lead changes, opposing fans’ behaviors, etc. Final limitation was that we neglected playing position differences.

Increased metabolic conditions, as witnessed by increasing HRs, decrease JS%. Therefore, players should be encouraged to rest as much as possible during game-play pauses, and coaches should support this resting strategy by calling timeouts and managing substitutions wisely, especially during the final minutes of close games. Players should perform tailored physical training to condition their physical capacity and technical skills simultaneously. Our findings, obtained by a means of a non-game-play protocol, should be taken into account with caution, even if they provide indications similar to those provided by similar studies on other basketball fundamentals. Future research could investigate the JS in greater depth in other age and sport experience groups, and could repeat our approach in investigating other team sports fundamentals (e.g., volleyball blocking).

## Conclusion

The findings of the present study have implications for professionals working with basketball players. Based on these results, it is recommended that coaches focus on exercises using the JS under metabolic conditions simulating a basketball game. Our results suggest two practical applications to maintain high JS%: (1) preliminary warm-up (even if needed to protect the body against injuries) does not improve JS accuracy, since the between 50HR and 0HR difference was not significant; and (2) 80HR decreases JS accuracy significantly, therefore providing scientific support for the usual behavior of players who always aim to rest as much as possible during game-play pauses, and for coaches, who support players’ behavior by calling timeouts and managing substitutions accordingly, especially during the final minutes of close games.

## Author Contributions

All authors listed have made a substantial, direct and intellectual contribution to the work, and approved it for publication.

## Conflict of Interest Statement

The authors declare that the research was conducted in the absence of any commercial or financial relationships that could be construed as a potential conflict of interest.

## Footnotes

1 BASKETBALL REFERENCE, http://www.basketball-reference.com/play-index/plus/shot_finder.cgi

2 A New View of Statistics, http://www.sportsci.org/resource/stats/
